# Molecular basis for avirulence of spontaneous variants of *Porphyromonas gingivalis*: Genomic analysis of strains W50, BE1 and BR1

**DOI:** 10.1111/omi.12373

**Published:** 2022-06-02

**Authors:** Joseph Aduse‐Opoku, Susan Joseph, Deirdre A Devine, Philip D Marsh, Michael A Curtis

**Affiliations:** ^1^ Centre for Host‐Microbiome Interactions Faculty of Dentistry Oral & Craniofacial Sciences, King's College London London UK; ^2^ Division of Oral Biology School of Dentistry University of Leeds Leeds UK

**Keywords:** collinearity, DNA inversion, genomic variants, haemin, heterogeneity, *Porphyromonas gingivalis*

## Abstract

The periodontal pathogen *Porphyromonas gingivalis* is genetically heterogeneous. However, the spontaneous generation of phenotypically different sub‐strains has also been reported. McKee et al. (1988) cultured *P. gingivalis* W50 in a chemostat during investigations into the growth and properties of this bacterium. Cell viability on blood agar plates revealed two types of non‐pigmenting variants, W50 beige (BE1), and W50 brown (BR1), in samples grown in a high‐hemin medium after day 7, and the population of these variants increased to approximately 25% of the total counts by day 21. W50, BE1 and BR1 had phenotypic alterations in pigmentation, reduced protease activity and haemagglutination and susceptibility to complement killing. Furthermore, the variants exhibited significant attenuation in a mouse model of virulence. Other investigators showed that in BE1, the predominant extracellular Arg‐gingipain was RgpB, and no reaction with an A‐lipopolysaccharide‐specific MAb 1B5 (Collinson et al., 1998; Slaney et al., 2006). In order to determine the genetic basis for these phenotypic properties, we performed hybrid DNA sequence long reads using Oxford Nanopore and the short paired‐end DNA sequence reads of Illumina HiSeq platforms to generate closed circular genomes of the parent and variants. Comparative analysis indicated loss of intact *kgp* in the 20 kb region of the *hagA‐kgp* locus in the two variants BE1 and BR1. Deletions in *hagA* led to smaller open reading frames in the variants, and BR1 had incurred a major chromosomal DNA inversion. Additional minor changes to the genomes of both variants were also observed. Given the importance of Kgp and HagA to protease activity and haemagglutination, respectively, in this bacterium, genomic changes at this locus may account for most of the phenotypic alterations of the variants. The homologous and repetitive nature of *hagA* and *kgp* and the features at the inverted junctions are indicative of specific and stable homologous recombination events, which may underlie the genetic heterogeneity of this species.

## 1 INTRODUCTION

Periodontitis is an inflammatory disease of the tooth‐supporting structure that can progress to connective tissue damage, loss of alveolar bone support and eventually tooth loss. The sequelae of events are a reflection of imbalanced host–microbiome homeostasis, preponderance of gram‐negative bacteria, and a dysbiotic community structure of the sub‐gingival plaque (Curtis et al., 2011, 2020; Holt & Ebersole, 2005). Central to the aetiology is the keystone pathogen *Porphyromonas gingivalis*; the bacterium is generally present in low abundance in health but increases in proportion in disease and is capable of initiating periodontal disease in experimental animal models (Hajishengallis et al., 2011; Payne et al., 2019). Furthermore, periodontitis is also linked to extra‐oral conditions including cardiovascular disease, diabetes, rheumatoid arthritis and Alzheimer's disease (Dominy et al., 2019; Sedghi et al., 2021). Hence, a comprehensive understanding of the mechanisms of pathogenesis associated with this bacterium may be important for the development of intervention strategies.

*Porphyromonas gingivalis*, a gram‐negative anaerobe, possesses an arsenal of virulence factors, including Arg‐ and Lys‐gingipain proteases, and other accessory proteins required for acquiring iron via haemin (Hajishengallis & Lamont, 2012; Lunar Silva & Cascales, 2021). On blood agar plates, *P. gingivalis* colonies are typically black. This characteristic phenotype, due to the ability to accumulate μ‐oxo bishaem, correlates with its virulence potential in a mouse model of virulence (Curtis et al., 2002). During experiments investigating the effects of growth conditions on *P. gingivalis* W50 in chemostats (McKee et al., 1988), the authors noted the appearance of colonial variants on blood agar plates following purity checks. The populations of these variants positively correlated with the duration of the initial chemostat experiments, that is, longer runs produced more non‐pigmented colonies. Furthermore, this phenomenon was only related to the inclusion of high haemin (iron protoporphyrin IX; 2.5 μg/ml) in the growth medium; the supplement is obligatory for the growth of *Porphyromonas* sp., and it is an essential source of iron. Two forms of the colonial variants, beige (BE1) and brown (BR1), were subsequently isolated and extensively characterised. The phenotypic changes in pigmentation were irreversible and stable. Furthermore, protease activity, agglutination of red blood cells, binding to haemin, and cell surface density in transmission electron microscopy were all dramatically reduced. In a mouse model of virulence, the variants were attenuated, with BE1 being the least virulent (Marsh et al., 1989; McKee et al., 1988; Smalley et al., 1989).

In the absence of gene‐targeted mutagenesis at the time, BE1 and BR1 were equivalent to mutants and were useful for comparative studies prior to the development of gene manipulation in *P. gingivalis*. For example, BE1 is rapidly killed by complement and lacks the anionic polysaccharide of the A‐lipopolysaccharide (A‐LPS), a typical post‐translational adduct of the type IX secretion system cargo proteins (Curtis et al., 1996; Slaney et al., 2006). However, the specific genomic changes in these variants have not been examined.

In this communication, we aim to characterise the genomic changes that may underlie the phenotypes of these spontaneous avirulent/pigment‐less variants of *P. gingivalis* W50 by comparative analysis of complete genomes. The genetic differences in the BE1 and BR1, which in part explain the major defects, are due to smaller *hagA* and complete loss of *kgp* and the intervening genes in the *hagA‐kgp* tandem loci; *hagA* encodes a major haemagglutinin required for agglutinating red blood cells, and *kgp* encodes the Lys‐gingipain protease. Furthermore, BR1 has incurred a major genome re‐arrangement, the breakpoints of which are outside coding genes.

## 2 MATERIALS AND METHODS

### 2.1 Growth of *P. gingivalis* W50, BE1 and BR1 strains

*Porphyromonas gingivalis* W50, BE1, BR1, K1A (Δ*kgp*) and PG0027 (Δ*porV*) cultures were resuscitated from microbead storage (Pro‐Lab diagnostic) stocks from −80°C on agar plates containing 5% (v/v) defibrinated horse blood (TCS Biosciences) in an atmosphere of nitrogen [N_2_] (80%), hydrogen [H_2_] (10%) and carbon dioxide [CO_2_] (10%) at 37°C (Don Whitley anaerobic work station) for 3 days and used within the next 5 days. When required, 24–48‐h liquid cultures were obtained by inoculating into 10 ml Brain Heart Infusion broth (Thermo Fisher) supplemented with 5 μg/ml haemin (Milner et al., 1996). The phenotypes of the strains were routinely confirmed by growing diluted samples on blood agar plates.

### 2.2 Arg‐ and Lys‐gingipain assays

The activities of Arg‐gingipains (RgpA and RgpB) and Lys‐gingipain (Kgp) in 48‐h grown broth cultures were assayed spectrophotometrically following the hydrolysis of N‐benzoyl‐DL‐arginine p‐nitroanilide and N‐α−acetyl‐L‐lysine‐p‐nitroanilide, respectively, at 30°C as described previously (Aduse‐Opoku et al., 1998; Rangarajan et al., 1997).

### 2.3 Whole‐genome sequencing and analysis

Genome sequencing of *P. gingivalis* W50, BE1 and BR1 strains was provided by MicrobesNG (http://www.microbesng.uk) using a parallel combination of Illumina (2 × 250 bp, paired‐end reads) and Oxford Nanopore (long reads) technologies on DNA samples extracted with an in‐house protocol to achieve > 30X coverage. Details of DNA extractions and pooled library preparations for DNA sequencing on Illumina HiSeq/NovaSeq or Oxford Nanopore technologies are presented at: https://microbesng.com/documents/24/MicrobesNG_Sequencing_Service_Methods_v20210419.pdf. Adapters were trimmed from Illumina sequences with Trimmomatic v0.30 and assembled with SPAdes v3.2. Trimmomatic was also used for Oxford Nanopore sequences generated on GridIon before assembly with Unicycler v0.4.0. The National Centre for Biotechnology Information (NCBI) accession numbers for *P. gingivalis* W50, *P. gingivalis* W50/BE1 and *P. gingivalis* W50/BR1 are CP092048, CP092049 and CP092050, respectively, under BioProject number PRJNA804142.

Data analysis and comparative genomics made additional use of other pipelines, such as NCBI (https://www.ncbi.nlm.nih.gov/) for analysis, comparisons and extraction of publicly available genomes, Artemis (Carver et al., 2005) for graphical viewing and extraction of sequence data, Progressive Mauve (Darling et al., 2010); http://darlinglab.org/mauve/download.html for graphical viewing and comparisons of whole‐genome data, RAST (Aziz et al., 2008), JGI (https://img.jgi.doe.gov/; Chen et al., 2019) for graphical viewing and systems comparisons, and BioEdit (Hall, 1999) for local viewing, extraction and analysis of sequence data.

## 3 RESULTS

### 3.1 Phenotypic and genotypic characterisation of *P. gingivalis* W50 variants BE1 and BR1

**FIGURE 1:**
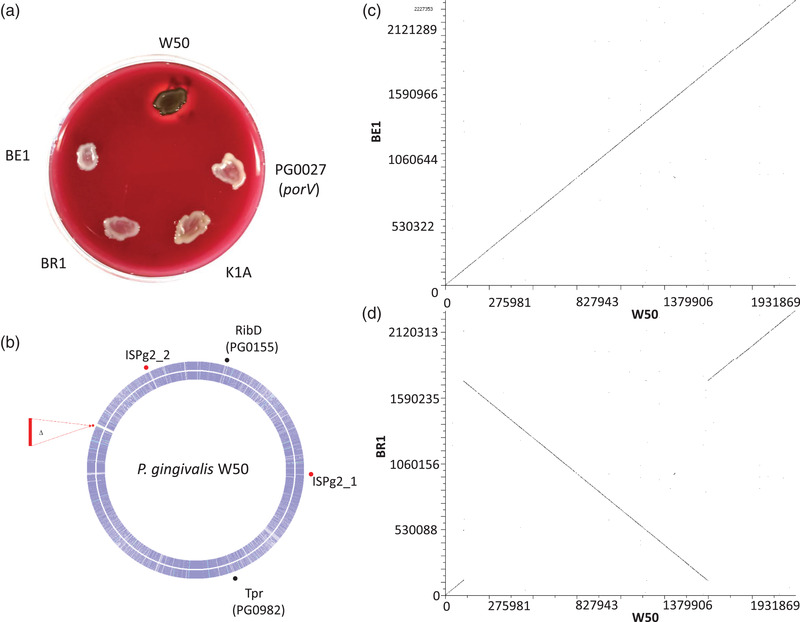
Phenotypic and genotypic comparisons of *Porphyromonas gingivalis* W50 with variants BE1, BR1, Δ*kgp* and Δ*porV*. *Porphyromonas gingivalis* cells were patched on blood agar plates, incubated anaerobically for 10 days and photographed (a); K1A and PG0027 represent Lys‐gingipain and type IX secretion system component (PorV) mutants, respectively. Whole‐genome alignments locate the positions of the deleted regions with reference to W50 in the centre outwardly followed by beige (BE1) and brown (BR1) (b). Genome locations represented by red dots are common to both BE1 and BR1, while black dots are specific to BE1 only. Diagon plots of the nucleotide sequence of W50 against BE1 (c) indicating collinearity or BR1 (d) highlighting the relative position of the major chromosomal DNA inversion in BR1. Note that the numbering of the genomes starts at the origin of replication, *dnaA*, in all strains

On blood agar plates, colonies of *P. gingivalis* W50 are phenotypically black due to its inherent ability to accumulate iron protoporphyrin IX. Furthermore, an extensive zone of haemolysis is also observed (Figure 1). However, in contrast, both variants have lost these properties and are unable to pigment and or haemolyse the blood in the agar. The BE1 and BR1 variants demonstrate a subtle difference; BE1 is white, whilst BR1 is straw‐coloured. These characteristics are reminiscent of the outcomes of targeted mutagenesis in either *kgp* (K1A) encoding Lys‐gingipain or components of the type 9 secretion system in *P. gingivalis* (Δ*porV*: PG0027 in Figure 1a encoding *porV*). The genetic basis of the phenotypic differences was further explored by determining the complete nucleotide sequences of the three strains. To achieve this, a hybrid combination of the long reads of Oxford Nanopore and the short paired‐end reads of Illumina platforms was used to obtain closed genomes. A summary of the results is shown in Table 1. Although the percentage guanine‐cytosine [% GC] (48.3) values are very similar to each other and *P. gingivalis* strains in general, there are subtle variations in genome sizes and gene contents, reflecting the general heterogeneity recognised within the species (Tables S1 and S2). Both variants are very similar to the parent exhibiting average nucleotide identities of > 99.99%, based on multiple sequence alignments (BLASTN, Table S1) as would be expected. Relative to the parent W50, the genomes of both BE1 and BR1 are smaller, indicating genome reductions or major loss of chromosomal DNA. This appears confined to similar locations, as indicated in the circular plot in Figure 1b. Despite the genome reductions, pairwise genomic comparisons indicate that BE1 is syntenic with W50; the genomes are collinear, while BR1 has undergone a major genome inversion (Figure 1c,d) involving a large section of the chromosome.

**TABLE 1:** Summary of the main genomic properties of *Porphyromonas gingivalis* W50, (beige) BE1 and brown (BR1)

**Strain**	**Genome size (bp)**	**GC (%)**	**Total number of genes**	**16S rRNA**	**23S rRNA**	**5S rRNA**	**tRNA**
*P. gingivalis* W50	2,345,841	48.3	2053	4	4	4	53
*P. gingivalis* W50 BE1	2,333,418	48.3	2049	4	4	4	53
*P. gingivalis* W50 BR1	2,332,345	48.3	2045	4	4	4	53

### 3.2 *Porphyromonas gingivalis* W50 is phylogenetically closer to strains W83 and A7436

**FIGURE 2:**
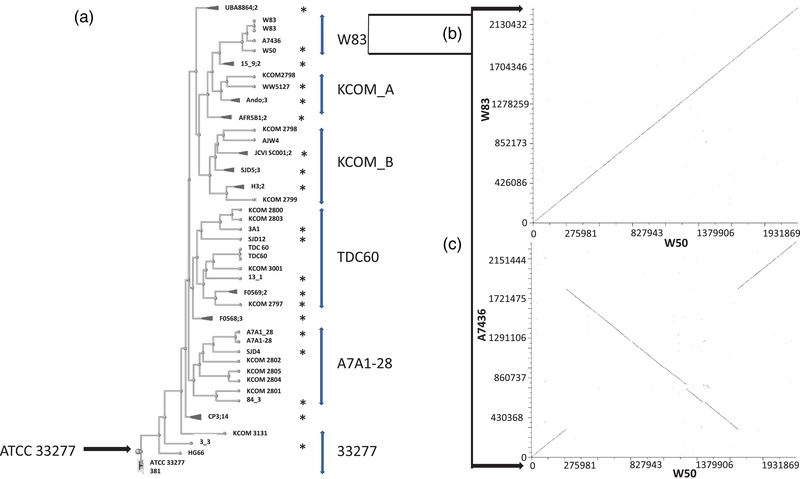
Comparison of *P. gingivalis* genomes from the National Centre for Biotechnology Information (NCBI). Phylogenetic analysis of 85 *P. gingivalis* genomes (a) and multiple sequence alignments of *P. gingivalis* W50 with the W83 clade (W83 and A7436 strains), shown as Diagon plots (b and c). The tree was rooted with reference to ATCC33277 (Naito et al., 2008). The original sources of these genomes are acknowledged in Table S2. The genomes in contig (22–192) forms are denoted with Asterix. Some of the branches (draft genomes) are collapsed to make the illustration clearer. The heterogeneities within the clades are shown as Diagon plots in Figure S1, where a representative is used to compare with each member

The 85 genomes of *P. gingivalis* deposited at the NCBI (https://www.ncbi.nlm.nih.gov/genome/tree/714), of which 22 are fully completed (Table S2), were used for comparison. In the phylogenetic tree, based on genome distances from CLUSTALW alignments and represented by the rectangular cladogram rooted against *P. gingivalis* ATCC33277, the genomes of W50, BE1 and BR1 cluster with *P. gingivalis* W83 (Nelson et al., 2003) and A7436 (Chastain‐Gross et al., 2015), in addition to the draft genome of *P. gingivalis* W50 from J. Craig Venter Institute, which has been deposited as 104 contigs (Figure 2a). Diagon plots, using the default parameters at NCBI, indicate that only one of the two W83 genomes (Nelson et al., 2003) is syntenic with W50 (Figure 2b) and maintains gene order. However, the newest W83 genome (Acuña‐Amador et al., 2018) shows DNA inversion relative to that of Nelson et al. This could potentially be an indication of a recent chromosomal aberration. However, the authors attributed this discrepancy to potential DNA sequence assembly issues in the earliest genome. The genome alignment of W50 and A7436 is, however, reminiscent of the W50/BR1 comparison; the breakpoint coordinates, and hence the chromosomal inversion, are distinctly different (308,926−1,831,349 bp; W50 numbering, Figure 2c). The first breakpoint corresponds to a non‐coding upstream region (on the bottom strand) of a gene encoding a hypothetical protein, whilst the second breakpoint is in a non‐coding region upstream (on the bottom strand) of a gene encoding uridine kinase (Udk). The consequence for *P. gingivalis* A7436 is loss of a mobile element (ISPg2 transposase), resulting in a 25 aa N‐terminal extension to its corresponding Udk; the biochemical and physiological significance requires further investigation. The breakpoints resulting in chromosomal inversion in A7436 do not exhibit any recognisable salient features. It is noteworthy that the copy number of the mobile element has been reduced from seven in W50 to four in A7436, indicating additional genome variations/re‐organisation. A second *udk* gene, smaller than the above and elsewhere in the genomes (1,870,343−1,870.972 bp; W50), is identical in size and sequence in both the W50 and A7436 strains. Comparisons of the other phylogenetic groups, using one member as the reference strain (x‐axis on graphs), to illustrate the significant heterogeneity in *P. gingivalis* strains are shown in Figure S1.

### 3.3 Both *hagA* and *kgp* loci are targets for chromosomal deletions in BE1 and BR1

**FIGURE 3:**
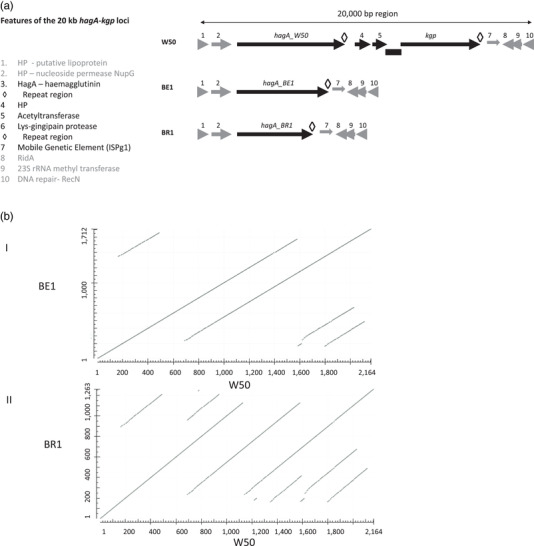
Genetic organisation of the *hagA‐kgp* loci in *P. gingivalis* W50, BE1 and BR1. (a) The putative functions of the genes shown in the schematic are listed. The chromosomal regions affected in BE1 and BR1 are in black. BE1 and BR1 have completely lost genes 4 (encoding hypothetical protein) and 5 (encoding acetyltransferase) in addition to *kgp* and its upstream sequences; smaller derivatives of HagA, exhibiting in‐frame internal deletions, are encoded in BE1 and BR1. The repeated regions encoding the C‐terminal domains of HagA and Kgp are represented by the symbol ◊ and are shown in detail in Figure S2. The genes surrounding the *hagA‐kgp* loci are unaffected (in grey). (b) Comparison of HagA_W50_ with HagA_BE1_ (I) or HagA_BR1_ (II)

Closer examination of the deleted regions (Figure 1b) in both BE1 and BR1 indicates that the genetic locus comprising a tandem *hagA* (upstream) and *kgp* (downstream), encoding HagA (haemagglutinin A) and Kgp (Lys‐gingipain), respectively, are majorly affected (Figure 3). In *P. gingivali*s W50, the genomic structure and organisation of the corresponding region encode HagA at the 5‘‐end preceding a hypothetical gene (4), acetyltransferase (5), three small genes (hypotheticals) and *kgp* at the 3′‐end. Further downstream is a mobile genetic element (ISPg1, 7) that has an internal termination and exists as two orfs; presumably, these are rendered non‐functional. HagA is a large protein of 2164 aa that shares repeated domains with Kgp, most notably the C‐terminal domain (Figure 3, ◊), which is an essential signature of cargo proteins of the type IX secretion systems in the Bacteroidetes phylum; the translated sequence encodes the carboxy‐terminal 70 aa (Figure S2). This is within the suggested 70–80 aa range (Glew et al., 2012) required for translocation of substrates to the cell's surface/external milieu by the type IX secretion system. However, in both BE1 and BR1, the *hagA‐kgp* loci are truncated to the point where only a derivative of *hagA*, encoding smaller versions of HagA are present; HagA_BE1 _= 1712 aa and HagA_BR1 _= 1263 aa (Figure S2). Thus, an event, probably involving homologous recombination, has occurred with the loss of DNA resulting in a concomitant remnant of a smaller *hagA*. This is accompanied by the loss of one of the two repeating regions, genes 4 (hypothetical) and 5 (acetyltransferase), in both variants at this locus. The regions juxtaposed to *hagA‐kgp* loci, including the interrupted transposase (ISPg1), are preserved in all three *P. gingivalis* strains; *kgp* is completely absent within the genomes of BE1 and BR1. The lack of Kgp enzymatic activities in BE1 and BR1 was confirmed by biochemical assays (not shown). Thus, functional *kgp* is completely absent from the variant genomes and not relocated elsewhere outside its normal locus.

### 3.4 Transposase copy number variations

*ISPg2* is another mobile element of the IS4 family of transposases; W50 possesses 7 copies of *ISPg2*, while both BE1 and BR1 are missing two copies at the expected locations (Figure S3); the variants have five copies each. The copy number variations of *ISPg2* in the three strains have already been alluded to with respect to strain A7436 (which possesses four copies). In the first case, there are no obvious deleterious defects around the locus. However, the second deleted ISPg2 in both BE1 and BR1 resulted in the remnant genes (IS195; ISPg3) encoding extralong and altered N‐termini (6 aa) from upstream sequences; that is, the *orfs* have gained additional sequences to extend their coding capacities. These regions are all part of numerous repeated regions in the genomes and encode mobile elements of the IS195 family of transposases (ISPg3). There were five completely identical copies of ISPg3 in the strains. The information above relates to an additional sixth copy. Thus, there is scope for multiple and perhaps simultaneous events to have occurred, resulting in DNA deletions and contributing to genome reductions (Table 1) and heterogeneity (Figure S1). In contrast, the 1131 bp *ISPg1* of *P. gingivalis* usually encodes a protein of 361 aa of the IS5 transposase family, and some forms are interrupted by premature termination, leading to two orfs (Figure 3, gene 7); the 26 copies of *ISPg1* in W50 align perfectly with those in BE1 and BR1.

### 3.5 A gene encoding a tetratricopeptide repeat is truncated at the 5′‐end in BE1

A 2253 bp region of the W50 genome (locus_tag MCS25_04475; 1,045,646−1,047,898 bp) encodes a 750 aa tetratricopeptide repeat protein; these are known to participate in a variety of cellular processes, including regulation and iron uptake (Morabe & McCarter, 2020). The gene is identical to BR1. However, in BE1, pairwise alignment between W50 and BE1 shows a perfect match except between regions 411–820 bp; the internal region of the gene at the 5′‐end in BE1 is deleted. Reminiscent of the *hagA‐kgp* locus in BE1 and BR1, the remnant of the locus encodes a 614 aa protein lacking A^27^–P^162^ of W50. Alternatively, since the region is highly repetitive, this could also be interpreted as a deletion in A^95^–A^231^ or I^119^–A^255^. These regions encode tetratricopeptide repeats that display multiple domain architectures that are important to the binding functions of this protein (Figure S4a,b).

### 3.6 Breakpoints at chromosomal DNA inversion in BR1 show complementary motifs

**FIGURE 4:**
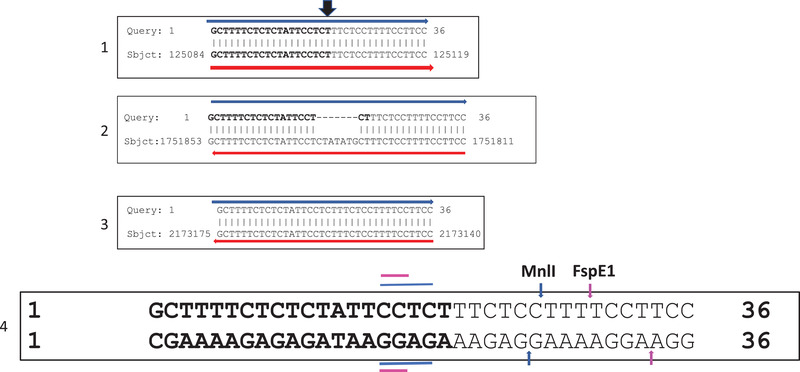
Characteristic nucleotide sequence within *P. gingivalis* W50 that are at the boundary sites of the inverted DNA in BR1 (Blocks 1 and 2) at the positions indicated. The homologous sequences in Blocks 2 and 3 are on the bottom (minus) strand, as indicated by the directions of the red and blue horizontal arrows. Hence, the sequences are complementary on the top (plus) strand. Block 3 is encoded by the complementary strand at the end of the gene *addA*, which encodes an ATP‐dependent helicase. The breakpoints at the junctions are shown above the sequences with the overhead black arrow (Block 1). The locations of the restriction enzyme recognition sites for MnlI and FspE1 are indicated in Block 4 with blue and pink arrows, respectively, with the tetranucleotide and dinucleotide core sequences as coloured horizontal bars.

Pairwise comparison of the genomes of *P. gingivalis* W50 and BR1 (Figure 1d) shows loss of synteny between positions 125,103 and 1,751,834 bp (wild‐type W50 numbering). However, the sequence is on the opposite (minus) strand in BR1, indicating chromosomal inversion in this variant. A characteristic feature of these junctions is the presence of a 36 bp sequence motif (Figure 4; box 1 and 2) with two prominent DNA restriction sites for enzymes MnlI and FspE1 (Figure 4; box 4). Although the recognition sequences are long, the small core signatures (dinucleotide and tetranucleotides) occur too frequently within the genomes to be of any significance. FspE1, however, requires methylated DNA, possibly adding another level of complexity to their phenotypes. At the 5′‐end in W50, the 36 bp sequence is situated immediately downstream of a 5S rRNA gene and upstream of *topB*, encoding DNA topoisomerase III. The same sequence at the 3′‐end is interrupted by an additional 7 nt (Figure 4; box 2), and it is upstream of a 23S rRNA gene within the vicinity of *tolC*, encoding an outer membrane lipoprotein. Furthermore, a complementary sequence of the 3′‐end of an *addA*, encoding an adenosine triphosphate [ATP]‐dependent helicase, forms the third area of the genome with sequence homology to the 36 bp signature (Figure 4; box 3); the sequence motif is present within an annotated gene and does not show any signs of chromosomal aberration in the variants. Since the 5′ and 3′‐ repeats (boxes 1 and 2) are complementary to each other, a scenario of DNA hybridisation, excision and repair possibly leading to chromosomal DNA inversion is a possibility.

### 3.7 RibD is different in BE1

The gene normally encodes a bifunctional (deamination and reduction) enzyme in the riboflavin biosynthetic pathway. This generates flavin mononucleotide and flavin adenine dinucleotide cofactors for intermediary metabolism. The genes in both W50 and BR1 are 100% identical (Figure S5). However, two additional guanosine residues ‘GG’ at the 3′‐end in BE1 lead to a different C‐terminus, suggesting that RibD could contribute to the subtle phenotypic difference between BR1 and BE1. To attempt to address this, we embarked on the complementation of *ribD*_BE1_ with *ribD*_W50_. Initially, *ribD* was insertionally inactivated with an *erm* cassette in all strains. The mutants were extremely slow to grow on blood agar plates; normal growth could not be restored by riboflavin supplementation as is the case in *Vibrio cholerae* (Fuentes Flores et al., 2017), and further genetic manipulation was not feasible. Therefore, *ribD* in *P. gingivalis* may have a different role independent of riboflavin biosynthesis. In *Mycobacterium tuberculosis*, the RibD homologue is not involved in riboflavin biosynthesis (Cheng & Sacchettini, 2016).

### 3.8 Examination of other virulence factors

#### 3.8.1 Arg‐gingipains A and B *(rgpA* and *rgpB*)

The gene organisation and localities of *rgpA* are identical in all three strains; *rgpA* encodes proteins of 1706 aa demonstrating subtle variations in molecular weights (W50 = 185656.66 Da; BE1 = 185674.56 Da; BR1 = 185674.56 Da). This is due to the effects of a 2 aa change, including one that is synonymous; K^1088^ and A^1990^ of W50 are substituted with E^1088^ and S^1990^, respectively, in both BE1 and BR1. These changes are within the haemagglutinin domains of RgpA and therefore are not expected to compromise the enzymatic activities of the catalytic domains.

The genes encoding the 706 aa of RgpB and its localities (on the inverted DNA of BR1) are identical in sizes of genes, order and organisation. Thus, a possible explanation for the reduced Arg‐gingipain activities in BE1 and BR1 could not be found, structurally and topologically, and therefore other factors are likely to contribute to their apparent reduction in Arg‐gingipain protease activities.

In addition, we interrogated the *P. gingivalis* genomes and compared other documented virulence factors/haemin‐related genes and loci for sequences and organisation. However, we were unable to demonstrate differences in loci including *rag*A/B, capsular polysaccharide, *porR* locus and other genes involved in LPS biosynthesis, such as PG1964 (*wbaP*, *wecA*), PG1051 (A‐, O‐antigen ligase), PG2119 (*wbpB*), PG0129 (mannosidase) and PG0027 (*porV*, *lptO*).

## 4 DISCUSSION

Isolation of the two main colonial variants of *P. gingivalis* W50, BE1 and BR1 from chemostat grown cells and subsequent characterisation in terms of association with virulence and loss of biochemical, physiological and structural properties were instrumental in the variants being synonymous with mutants; the mutations were locked, irreversible and permanent (Marsh et al., 1989; McKee et al., 1988; Slaney et al., 2006; Smalley et al., 1989). Although the specific defects were unknown, these variants were extremely useful for comparative studies investigating the potential mechanisms used by *P. gingivalis* in the pathogenesis of periodontitis. *Porphyromonas gingivalis* isogenic mutants, generated by gene‐targeted mutagenesis (Fletcher et al., 1995), emerged several years later to assist in finely defining virulence factors, while the molecular basis of virulent to avirulent conversion of *P. gingivalis* W50 was largely ignored. Chen and Kuramitsu (1999) noted the spontaneous appearance of pigment‐less colonies from their defined *P. gingivalis* 381 mutants in *rgpA*, *rgpB* and *prtT* in a manner akin to those of BE1 and BR1 (McKee et al., 1988). The parent, however, was sturdy, and no apparent appearance of the colonial type was noted by repeated sub‐culturing, implying that the genomes instabilities are related to genetic manipulation. Further investigation, by targeted Southern hybridisation, suggested that the *hagA‐kgp* region of the genome may be subjected to homologous recombination, losing *kgp* and hence pigmentation in the process in all three strains. In *P. gingivalis* HG66 (Acuña‐Amador et al., 2018), the corresponding region possesses intact *hagA* and *kgp*. However, the region between the two genes has expanded and is riddled with remnants of both genes, suggestive of homologous recombination. Kgp is also required to bind and acquire haemin from red blood cells and to facilitate pigmentation (Lewis et al., 1999); the process is also dependent on A‐LPS (Rangarajan et al., 2017).

In this communication, we compared the complete genome sequences of *P. gingivalis* W50 and variants BE1 and BR1. We report that the genomes are phylogenetically similar to each other and also to *P. gingivalis* W83 and *P. gingivalis* A7436 (Figures 1, 2, 3; Tables 1, S1 and S2). This is in agreement with several other studies and confirms the genomic and physiological heterogeneity among *P. gingivalis* strains (Acuña‐Amador et al., 2018; Chen et al., 2017; Coats et al., 2019; Suwannakul et al., 2010).

Relative to *P. gingivalis* W50, the genomic aberrations in BE1 and BR1 are of two main types: deletions affecting both variants and chromosomal inversion affecting only BR1. The major deletion involved the *hagA‐kgp* loci (Figures 1 and 3) and suggests the possibility of DNA excision that would involve hybridisation, homologous recombination and loss of DNA. This mechanism seems precise, that is, the remnants are not nonsense genes but are derivatives of *hagA*, and in‐frame excision of DNA has occurred. Since *ha*gA and *kgp* are highly homologous genes, this mode of reaction is likely to be reliant on repeated sequences within the loci, including the 3′‐end of both genes that are virtually identical (Figure S2). The process is reminiscent of the observations of Chen and Kuramitsu (1999). In this case, an unknown trigger caused similar chromosomal aberrations in *P. gingivalis* mutants defective in *rgpA*, *rgpB* and *prtT* (variants MT10‐W, G102‐W and WK‐W, respectively) with accompanying deletions in the *hagA‐kgp* loci, also manifested as a reduced ability to agglutinate red blood cells, loss of haemolysis and lack of Kgp activity. Both *rgpA* and *rgpB*, encoding the Arg‐gingipains A and B, respectively, are highly homologous. Yet, there is no report of potential recombination between these two genes. Thus, in the case of *hagA‐kgp* loci, local topology and perhaps close gene proximity, encouraged by the physiological state of the cells, are essential factors for recombination/deletion. This then leads to ‘structural genotypes’ with major consequences on growth, physiology and biochemical properties (Page et al., 2020).

The Arg‐gingipain proteases of *P. gingivalis* comprise homologous RgpA and RgpB that are processed and posttranslationally modified with varying degrees of glycosylation to give five major isoforms. These are derived from two genetic loci. In *P. gingivalis* W50, the predominant Arg‐gingipain in the supernatant is derived from *rgpA* (Rangarajan et al., 2008). However, in *P. gingivalis* BE1, the total Arg‐gingipain activity was reduced to 25%−30%. In addition, the variant demonstrated improperly processed RgpB preprotein and lacked the heavy glycosylation typical of *P. gingivalis* W50 (Collinson et al., 1998). Thus, at the biochemical level, these reports partially confirm previous observations (Marsh et al., 1989; McKee et al., 1988; Smalley et al., 1989). However, our molecular analysis could not explain the reduction in Arg‐gingipain activity in either BE1 or BR1, as the sequences and loci are the same as those in W50.

The loss of two copies of the transposase ISPg2 (Figure S3a,b) in BE1 and BR1 is reminiscent of the above phenomenon involving *hagA‐kgp* loci. In the first case, there is no apparent deleterious effect; the excision of ISPg2 is neat and has no further apparent consequence. However, the second loss of ISPg2 resulted in the coding potential of the neighbouring IS195 (IS195*, ISPg3*) being extended to include an additional 6aa at the N‐terminus. Whether this is physiologically relevant remains to be shown. During the development of gene‐transfer methods for *P. gingivalis*, Maley and Roberts recovered pNJR12 derivatives from *P. gingivalis* W83 that were bigger than the original plasmid. Subsequent characterisation identified IS1126 (ISPg1) that had transposed onto the autonomously replicating shuttle plasmid (Maley & Roberts, 1994; Maley et al., 1992). Furthermore, Lewis et al. cloned and characterised *kgp* from *P. gingivalis* W83. One of the clones was isolated had *kgp* inactivated with IS195 (ISPg3; Lewis & Macrina, 1998). Both sequenced *P. gingivalis* W83 strains possess a gene (PG1739 and CF003_1739) that is annotated as encoding a PppX/GppA phosphatase of 193 aa. In *P. gingivalis* W50, BE1 and BR1, the protein is 302 aa. Close examination indicated that in *P. gingivalis* W83 PG1739 had undergone a precise, neat internal deletion, resulting in a smaller protein. This is likely to contribute to strain–strain variations and subtle phenotypic changes.

In BR1, the junctions at the large chromosomal DNA inversion are characterised by a 36 bp DNA sequences outside coding regions (Figures 1d and 4) that could also be subjected to pairing, annealing, hybridisation and recombination, resulting in DNA inversion and additionally contributing to genetic heterogeneity (Figures 2a and S1). However, a third copy of the repeated motif is also present at the 3′‐end of *addA*, encoding an ATP‐dependent helicase, without any noticeable deleterious effect. Thus, the reasons and significance of these genomic reactions are topics for further investigations.

Pigmentation of *P. gingivalis* on blood agar plates is generally due to three main factors: interference with Kgp activity, inability to synthesise anionic polysaccharide of the A‐LPS, and disruption to the type IX secretion system components (Curtis et al., 2002; Gallagher et al., 2003; Glew et al., 2012; Klein et al., 2017; Nakayama, 2015; Rangarajan et al., 2017; Shoji et al., 2002, 2014, 2018). The absence of *kgp* in the BE1 and BR1 variants is a prominent genotypic feature of the variants (Figure 3) and could alone explain the loss of pigmentation and attenuated virulence. Whether the subtle additional changes observed, such as the extent of deletion in the *hagA‐kgp* loci and/or the tetratricopeptide gene and RibD alterations in BE1, differentiate the two variants requires further investigation.

## CONFLICT OF INTEREST

The authors declare that there is no conflict of interest that could be perceived as prejudicing the impartiality of the research reported.

### PEER REVIEW

The peer review history for this article is available at https://publons.com/publon/10.1111/omi.12373.

## Supporting information

Supporting Information

## Data Availability

The data that support the findings of this study are openly available in GenBank at https://www.ncbi.nlm.nih.gov/, reference number PRJNA804142.
