# Supplementary material for: Molecular basis for avirulence of spontaneous variants of Porphyromonas gingivalis: Genomic analysis of strains W50, BE1 and BR1
Source: Mol Oral Microbiol. 2022 Jun 2;37(3):122–32. doi: 10.1111/omi.12373 (PMC9328147; doi:10.1111/omi.12373)
Supplement: Supplementary file 1 — Supporting Information [file OMI-37-122-s001.pptx]

## Slide 1
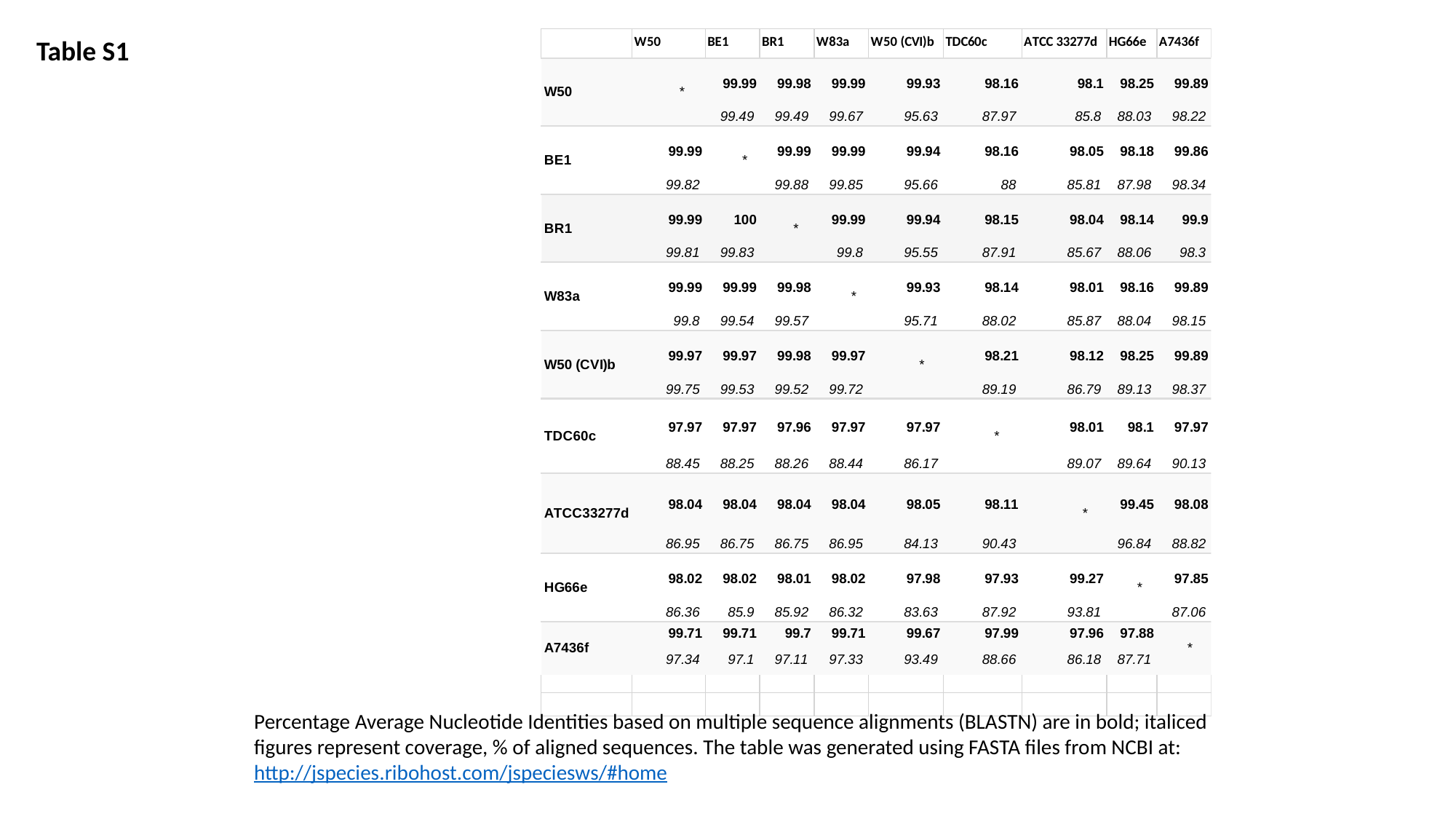

Table S1
Percentage Average Nucleotide Identities based on multiple sequence alignments (BLASTN) are in bold; italiced figures represent coverage, % of aligned sequences. The table was generated using FASTA files from NCBI at: http://jspecies.ribohost.com/jspeciesws/#home

## Slide 2
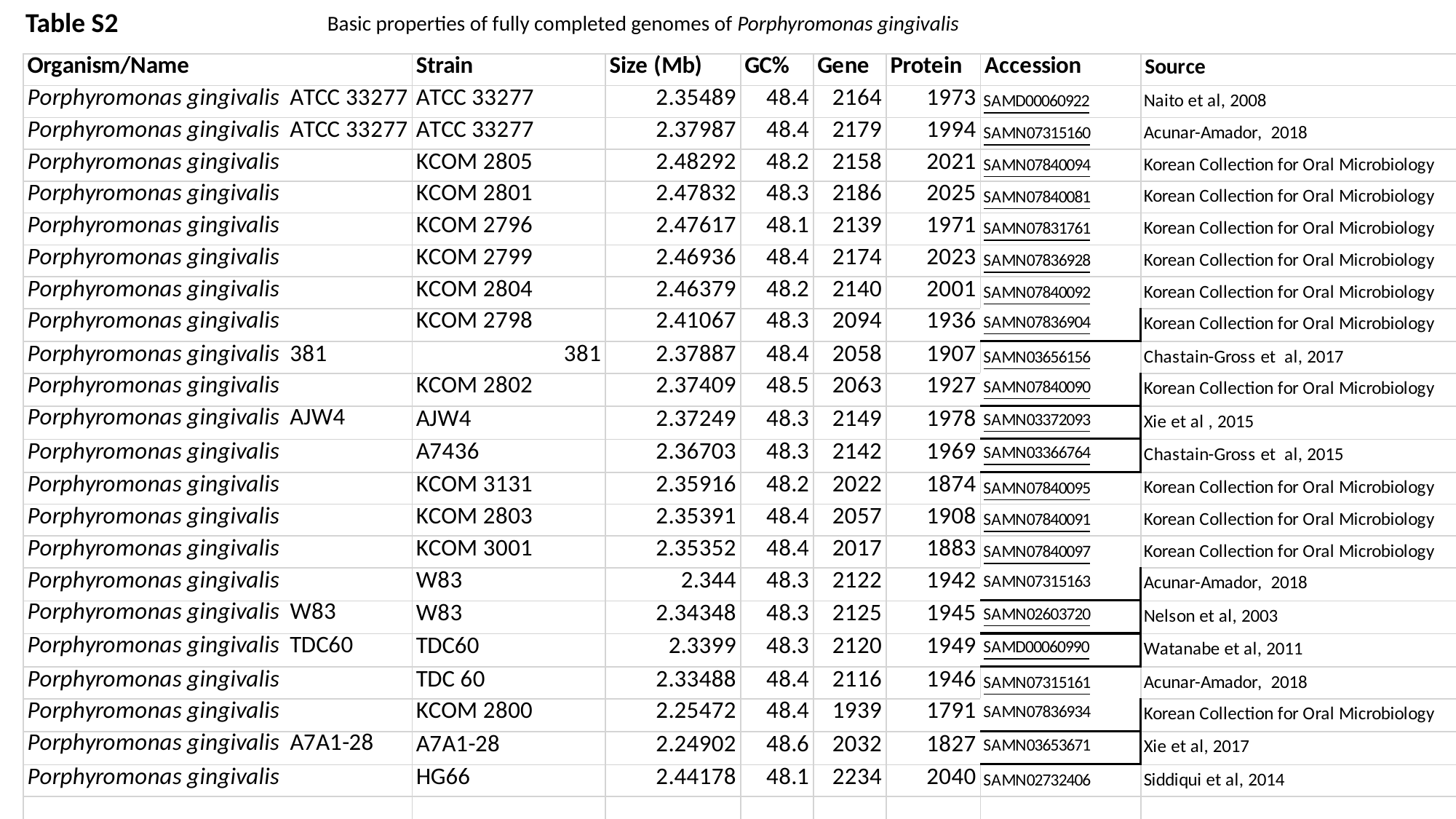

Table S2
Basic properties of fully completed genomes of Porphyromonas gingivalis

## Slide 3
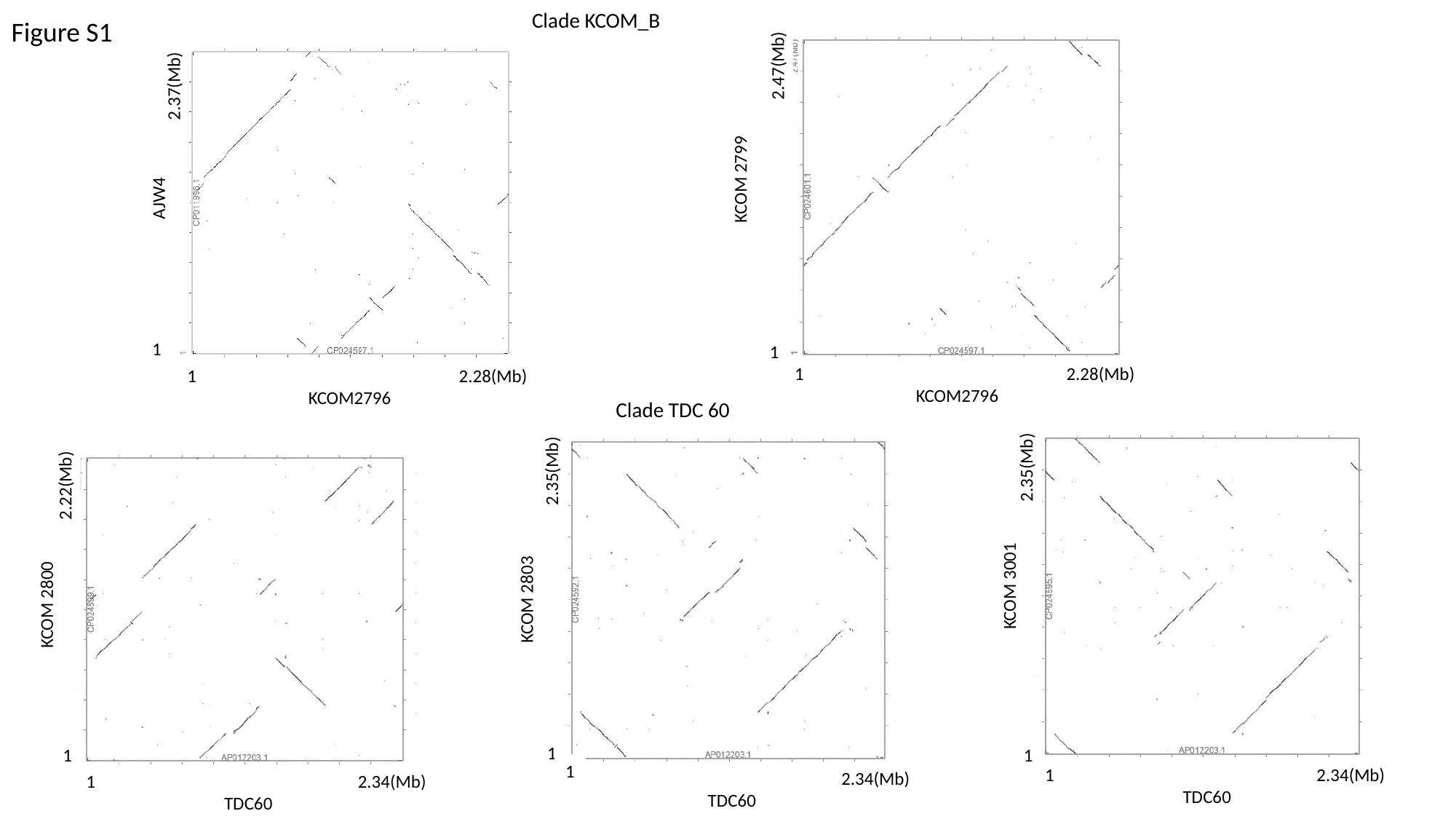

Clade KCOM_B
Figure S1
2.47(Mb)
2.37(Mb)
KCOM 2799
AJW4
1
1
1 2.28(Mb)
 KCOM2796
1 2.28(Mb)
 KCOM2796
Clade TDC 60
2.35(Mb)
2.35(Mb)
2.22(Mb)
KCOM 3001
KCOM 2803
KCOM 2800
1
1
1
1
1 2.34(Mb)
 TDC60
1 2.34(Mb)
 TDC60
1 2.34(Mb)
 TDC60

## Slide 4
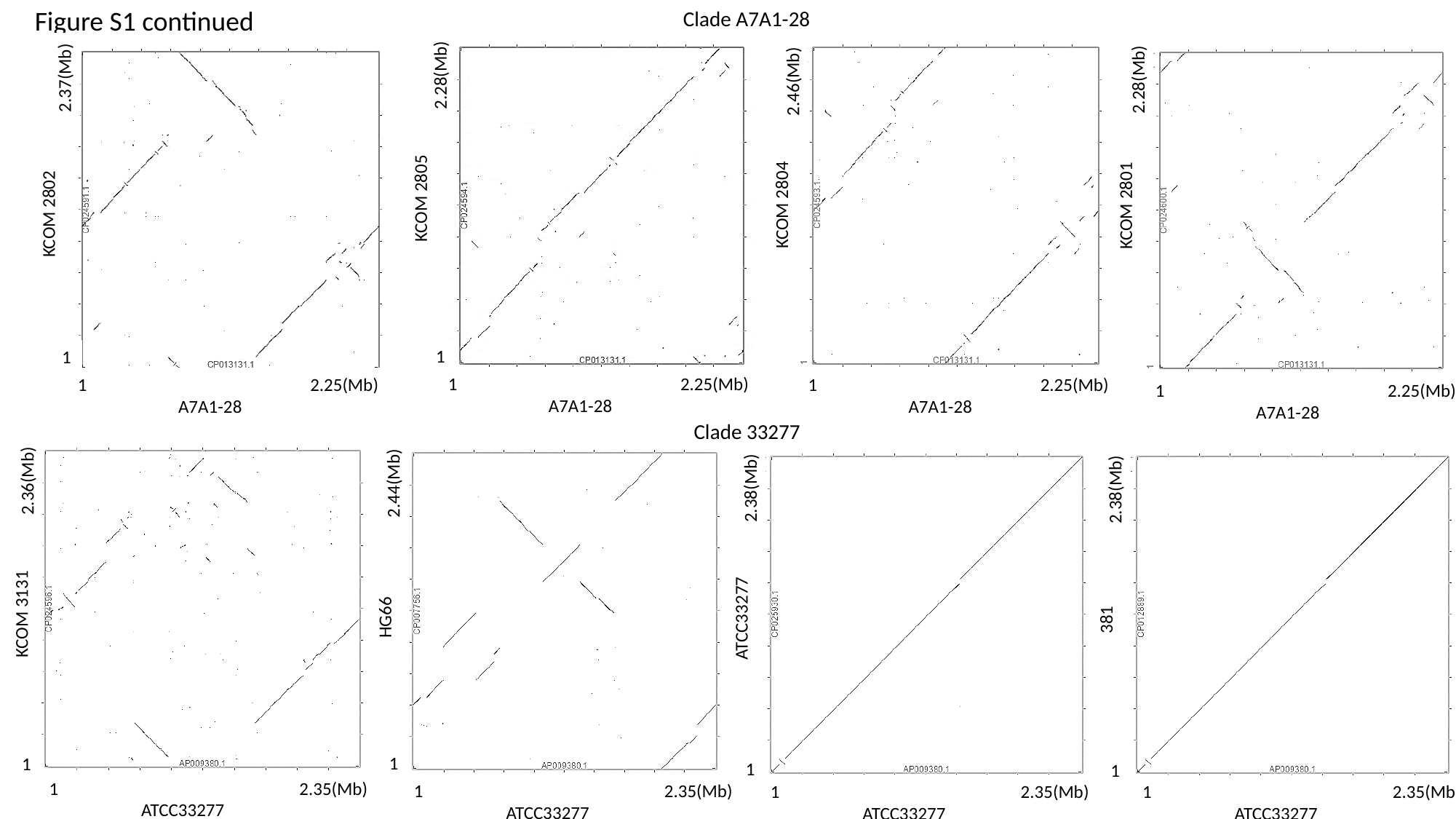

Figure S1 continued
Clade A7A1-28
2.28(Mb)
2.37(Mb)
2.28(Mb)
2.46(Mb)
KCOM 2805
KCOM 2804
KCOM 2801
KCOM 2802
1
1
 2.25(Mb)
 A7A1-28
 2.25(Mb)
 A7A1-28
 2.25(Mb)
 A7A1-28
 2.25(Mb)
 A7A1-28
Clade 33277
2.36(Mb)
2.44(Mb)
2.38(Mb)
2.38(Mb)
KCOM 3131
HG66
ATCC33277
381
1
1
1
1
 2.35(Mb)
 ATCC33277
 2.35(Mb)
 ATCC33277
 2.35(Mb)
 ATCC33277
 2.35(Mb)
 ATCC33277
 2.35(Mb)
 ATCC33277

## Slide 5
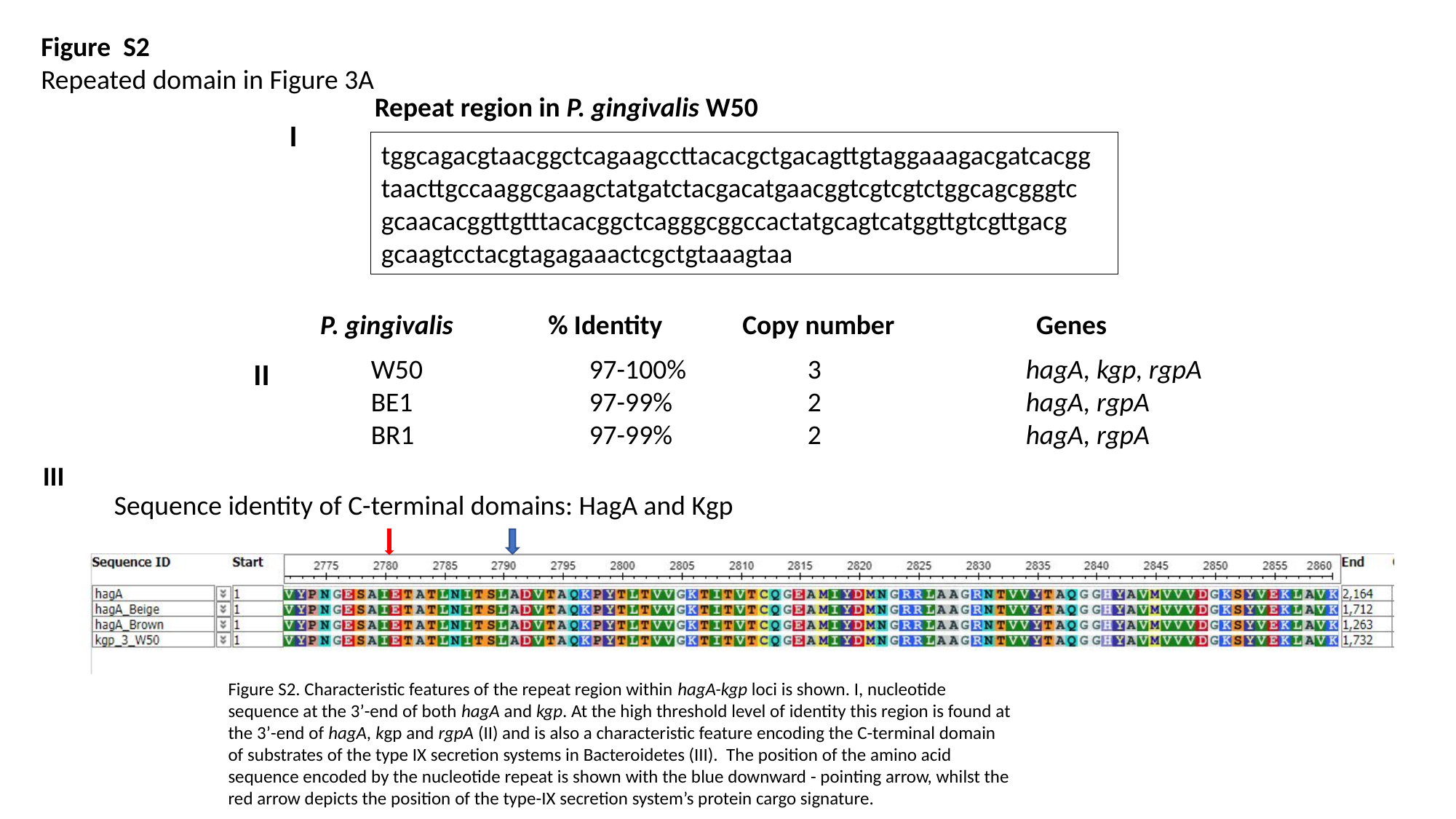

Figure S2
Repeated domain in Figure 3A
Repeat region in P. gingivalis W50
I
tggcagacgtaacggctcagaagccttacacgctgacagttgtaggaaagacgatcacgg
taacttgccaaggcgaagctatgatctacgacatgaacggtcgtcgtctggcagcgggtc
gcaacacggttgtttacacggctcagggcggccactatgcagtcatggttgtcgttgacg
gcaagtcctacgtagagaaactcgctgtaaagtaa
P. gingivalis
% Identity
Copy number
Genes
W50		97-100%		3		hagA, kgp, rgpA
BE1		97-99%		2		hagA, rgpA
BR1		97-99%		2		hagA, rgpA
II
III
Sequence identity of C-terminal domains: HagA and Kgp
Figure S2. Characteristic features of the repeat region within hagA-kgp loci is shown. I, nucleotide sequence at the 3’-end of both hagA and kgp. At the high threshold level of identity this region is found at the 3’-end of hagA, kgp and rgpA (II) and is also a characteristic feature encoding the C-terminal domain of substrates of the type IX secretion systems in Bacteroidetes (III). The position of the amino acid sequence encoded by the nucleotide repeat is shown with the blue downward - pointing arrow, whilst the red arrow depicts the position of the type-IX secretion system’s protein cargo signature.

## Slide 6
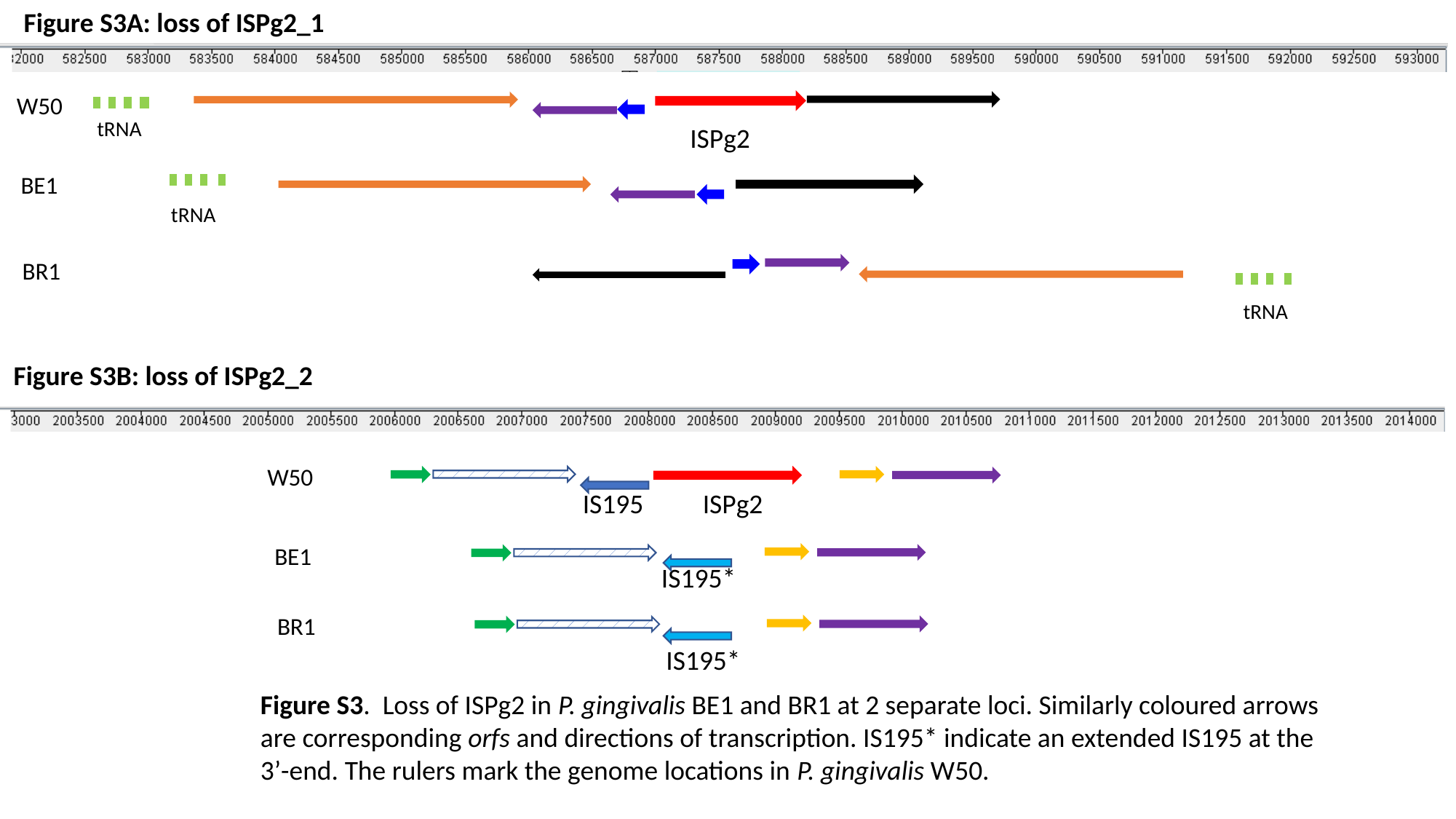

Figure S3A: loss of ISPg2_1
W50
tRNA
ISPg2
BE1
tRNA
BR1
tRNA
Figure S3B: loss of ISPg2_2
W50
IS195
ISPg2
BE1
IS195*
BR1
IS195*
Figure S3. Loss of ISPg2 in P. gingivalis BE1 and BR1 at 2 separate loci. Similarly coloured arrows are corresponding orfs and directions of transcription. IS195* indicate an extended IS195 at the 3’-end. The rulers mark the genome locations in P. gingivalis W50.

## Slide 7
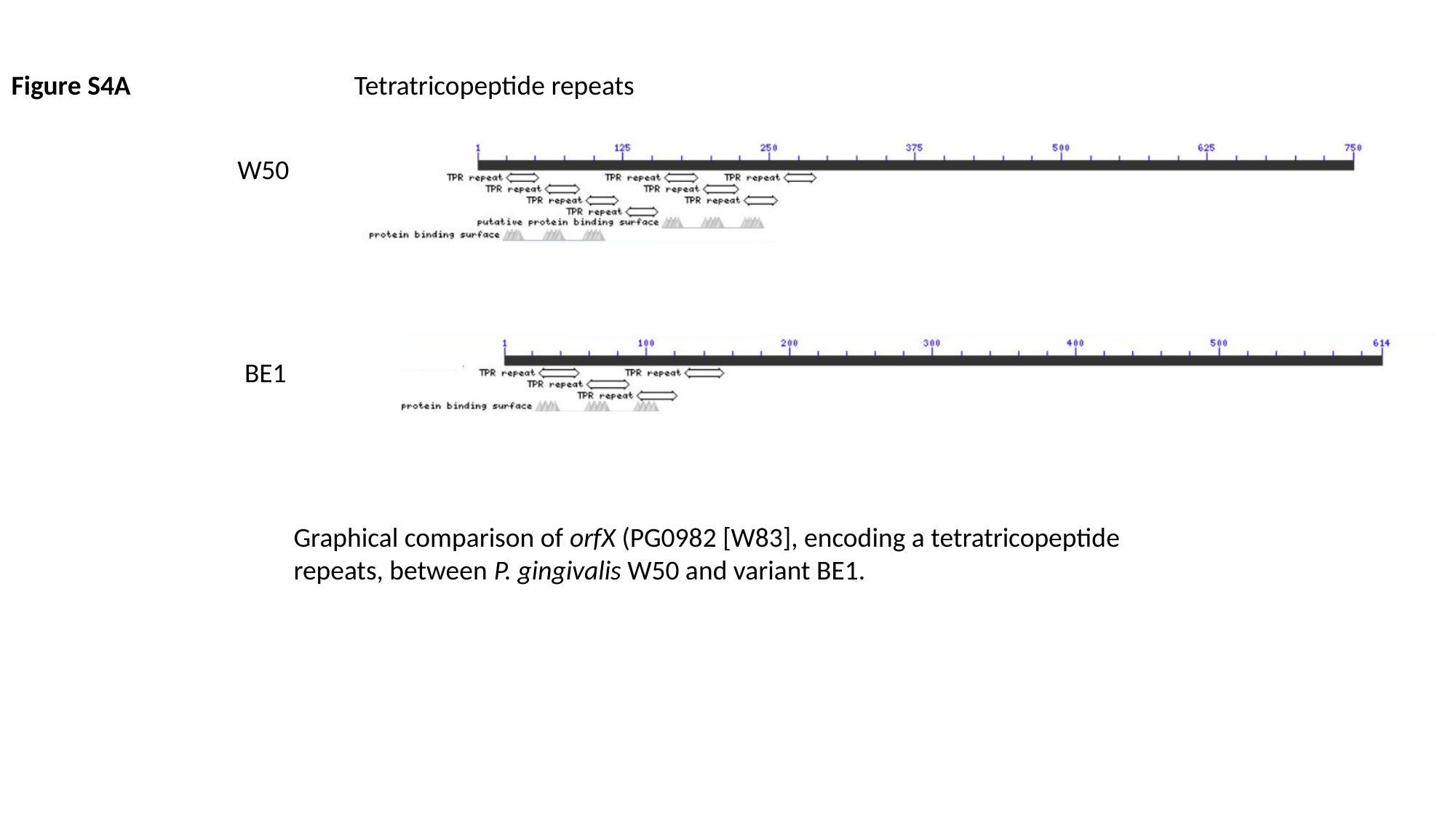

Figure S4A
Tetratricopeptide repeats
W50
BE1
Graphical comparison of orfX (PG0982 [W83], encoding a tetratricopeptide repeats, between P. gingivalis W50 and variant BE1.

## Slide 8
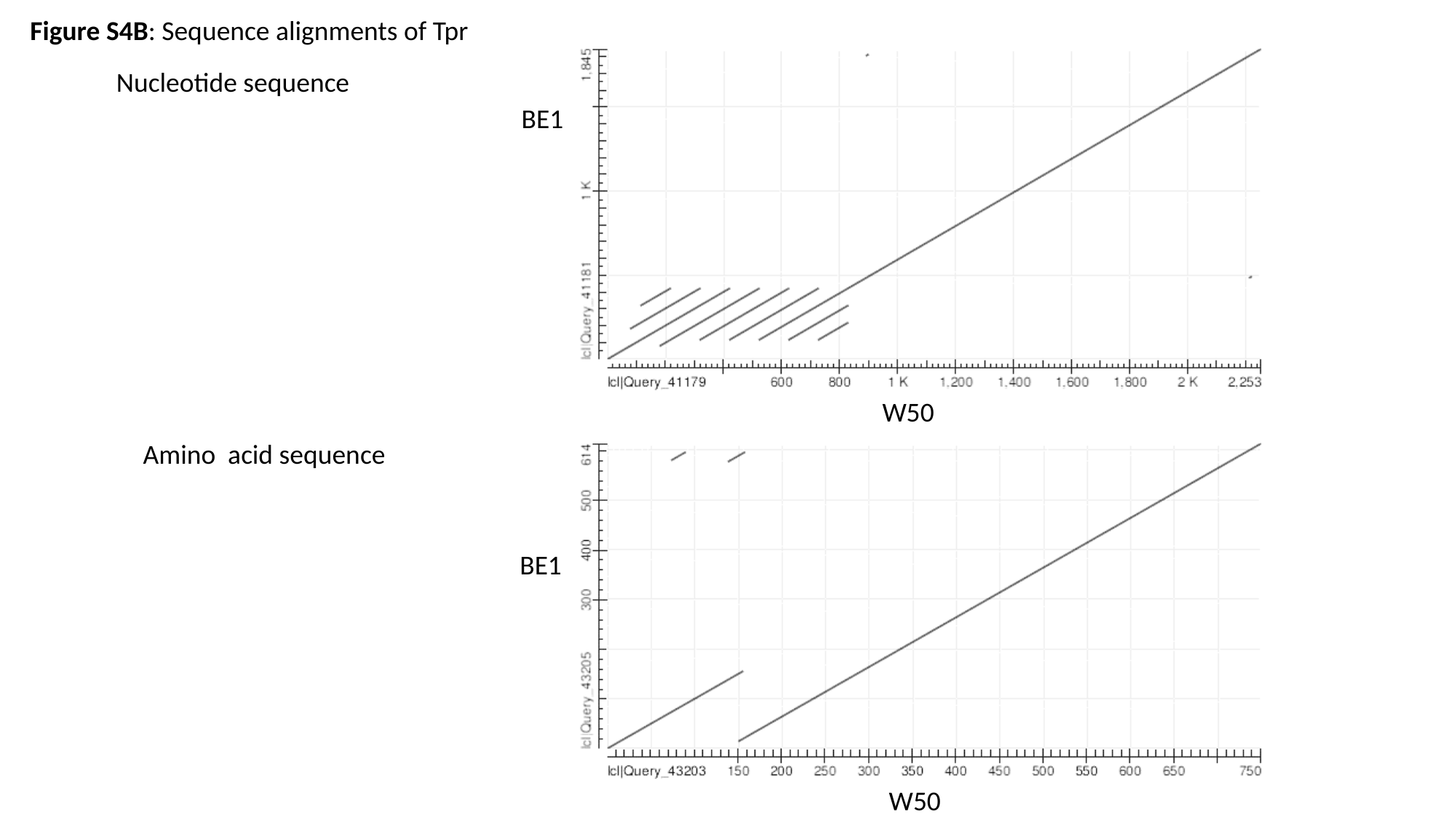

Figure S4B: Sequence alignments of Tpr
Nucleotide sequence
BE1
W50
Amino acid sequence
BE1
W50

## Slide 9
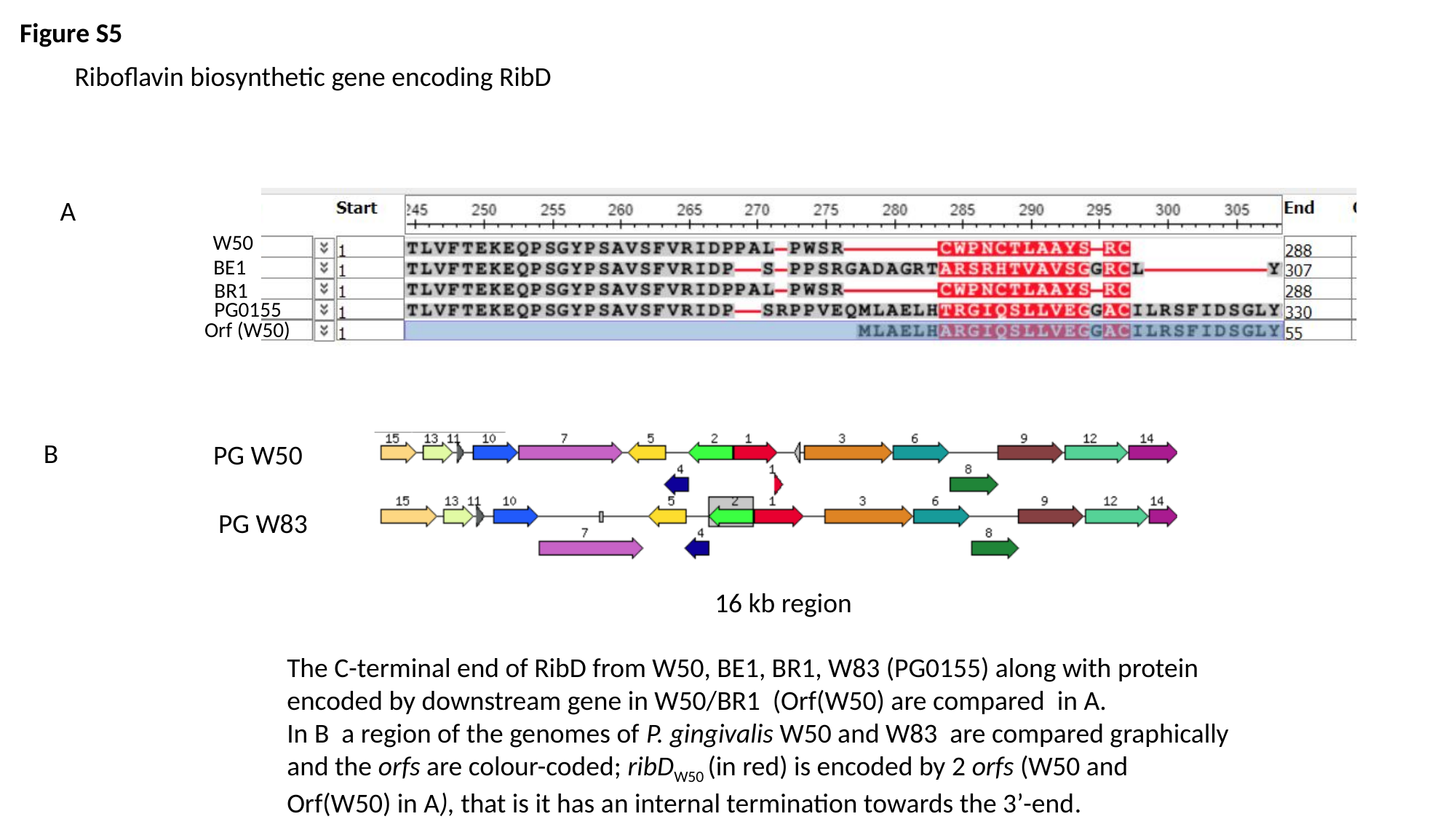

Figure S5
Riboflavin biosynthetic gene encoding RibD
A
W50
BE1
BR1
PG0155
 Orf (W50)
B
PG W50
PG W83
16 kb region
The C-terminal end of RibD from W50, BE1, BR1, W83 (PG0155) along with protein encoded by downstream gene in W50/BR1 (Orf(W50) are compared in A.
In B a region of the genomes of P. gingivalis W50 and W83 are compared graphically and the orfs are colour-coded; ribDW50 (in red) is encoded by 2 orfs (W50 and Orf(W50) in A), that is it has an internal termination towards the 3’-end.
